# Alloying Driven Antiferromagnetic Skyrmions on NiPS_3_ Monolayer: A First‐Principles Calculation

**DOI:** 10.1002/advs.202401048

**Published:** 2024-04-22

**Authors:** Yanxia Wang, Jianpei Xing, Ying Zhao, Yi Wang, Jijun Zhao, Xue Jiang

**Affiliations:** ^1^ Key Laboratory of Materials Modification by Laser Ion and Electron Beams Dalian University of Technology Ministry of Education Dalian 116024 China; ^2^ Guangdong Provincial Key Laboratory of Quantum Engineering and Quantum Materials School of Physics South China Normal University Guangzhou 510006 China; ^3^ Guangdong‐Hong Kong Joint Laboratory of Quantum Matter Frontier Research Institute for Physics South China Normal University Guangzhou 510006 China

**Keywords:** 2D NiPS_3_, alloying, antiferromagnetic skyrmions, density functional theory calculations

## Abstract

Topological magnetic states are promising information carriers for ultrahigh‐density and high‐efficiency magnetic storage. Recent advances in two‐dimensional (2D) magnets provide powerful platforms for stabilizing various nanometer‐size topological spin textures within a wide range of magnetic field and temperature. However, non‐centrosymmetric 2D magnets with broken inversion symmetry are scarce in nature, making direct observations of the chiral spin structure difficult, especially for antiferromagnetic (AFM) skyrmions. In this work, it is theoretically predicted that intrinsic AFM skyrmions can be easily triggered in XY‐type honeycomb magnet NiPS_3_ monolayer by alloying of Cr atoms, due to the presence of a sizable Dzyaloshinskii–Moriya interaction. More interestingly, the diameter of the AFM skyrmions in Ni_3/4_Cr_1/4_PS_3_ decreases from 12 to 4.4 nm as the external magnetic field increases and the skyrmion phases remain stable up to an external magnetic field of 4 T. These results highlight an effective strategy to generate and modulate the topological spin texture in 2D magnets by alloying with magnetic element.

## Graphical abstract

The intrinsic antiferromagnetic skyrmions can be easily triggered in XY‐type magnet NiPS_3_ monolayer by alloying of Cr atoms, due to the presence of a sizable Dzyaloshinskii‐Moriya interaction. More interestingly, the diameter decreases from 12 to 4.4 nm as the external magnetic field increases and the skyrmion phases remain stable up to an external magnetic field of 4 T.

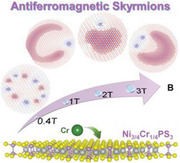

## 1 Introduction

Magnetic skyrmions,^[^ ^1^, ^2^, ^3^ ^]^ a kind of topologically protected whirling spin textures, have attracted enormous attention due to their potential applications in information memory and computing devices. Since 2009, numerous skyrmion hosting topological materials have been found,^[^ ^2^, ^3^, ^4^, ^5^, ^6^, ^7^, ^8^, ^9^, ^10^ ^]^ such as Bloch‐type skyrmions in bulk MnSi, Fe_0.5_Co_0.5_Si thin film, and helimagnetic FeGe thin films, antiskyrmions in Mn_1.4_Pt_0.9_Pd_0.1_Sn and Fe_1.9_Ni_0.9_Pd_0.2_P thin films, Néel‐type skyrmions in ferromagnetic (FM) multilayers, antiferromagnetic (AFM) skyrmions in AFM multilayers, and AFM half skyrmions in an AFM α‐Fe_2_O_3_ capped with Pt. However, there are still many limitations for the practical applications of skyrmions, such as extremely narrow window of stable region, large skyrmion size, and harsh fabrication technology.

The discovery of intrinsic long‐range magnetic ordering in two‐dimensional (2D) materials, such as CrI_3_, Cr_2_Ge_2_Te_6_, Fe_3_GeTe_2_, and MPS_3_ (M = Ni, Mn, Fe, Co),^[^ ^11^, ^12^, ^13^, ^14^ ^]^ provides new platforms for studying topologically nontrivial spin phenomena. Recently, many experimental and theoretical works have shown that dimensional reduction is capable of generating various types of small‐size magnetic skyrmions. Experimentally, Bloch‐type skyrmions have been observed in exfoliated van der Waals (vdW) materials Cr_2_Ge_2_Te_6_, whose diameter decreases from 120 nm at 11.7 mT to 77 nm at 195.8 mT.^[^ ^15^ ^]^ Room‐temperature clockwise and counterclockwise Bloch‐type magnetic skyrmions were also observed in layered Cr_1+x_Te_2_.^[^ ^16^ ^]^ In 2D Fe_3_GeTe_2_ sheet, Bloch‐type skyrmions would transform into skyrmion bubbles with increasing magnetic field and the average width of skyrmions is ≈120 nm.^[^ ^17^ ^]^ Néel‐type skyrmions with a size of ≈150 nm at 94 K and ≈80 nm at 198 K have also been observed in Fe_3_GeTe_2_‐based heterostructures.^[^ ^18^, ^19^ ^]^ Theoretically, various strategies have been proposed to further break the inversion symmetry of 2D magnets and stabilize the chiral spin textures. For instance, a field‐controlled Néel‐type skyrmion‐ferromagnet transition cycle has been predicted in a CrTe_2_/WTe_2_ heterostructure, in which the diameter of skyrmions is ≈20 nm.^[^ ^20^ ^]^ The transformation of bimeron‐skyrmions was realized via perpendicular strain and electric field in CrISe/In_2_Se_3_ heterostructure, the diameter of skyrmions is ≈14 nm.^[^ ^21^ ^]^ Néel‐type skyrmions with diameter of ≈10 nm were obtained in Janus CrInX_3_ (X = Te, Se), and the skyrmion phases can be sustained up to ≈180 K.^[^ ^22^ ^]^ In addition, skyrmion states and bimerons have also been detected in Janus MnXY (X, Y = S, Se, Te) and Cr(I, X)_3_ (X = Br, Cl) monolayers.^[^ ^23^, ^24^ ^]^ However, most of the aforementioned systems host FM skyrmions, which are sensitive to stray magnetic fields. In contrast to FM skyrmions,^[^ ^25^ ^]^ AFM skyrmions are a type of topological object consisting of similar but opposite spin texture on each sublattice, which have the advantage of the absence of stray magnetic fields and ultrafast dynamics.^[^ ^26^, ^27^ ^]^ AFM skyrmions‐based current‐driven logical gates and energy‐efficient diodes devices have been developed experimentally.^[^ ^28^, ^29^, ^30^ ^]^ Therefore, they offer greater potential for designing future nonvolatile logic computing devices with ultra‐low energy consumption and high‐density in AFM spintronics.

In principle, magnetic skyrmions are mainly stabilized by the competition between the antisymmetric Dzyaloshinskii–Moriya interaction (DMI) and the symmetric Heisenberg exchange (*J*).^[^ ^31^ ^]^ A negative *J* value denotes that AFM coupling between magnetic ions. DMI originates from spin–orbit coupling (SOC) and broken inversion symmetry. DMI and *J* favor canted and collinear alignments between neighboring spins, respectively. Experimentally, large DMI has been observed in ultrathin films epitaxially grown on heavy metal substances.^[^ ^32^, ^33^ ^]^ Moreover, large in‐plane magnetic anisotropy also favors the formation of topological defects.^[^ ^34^, ^35^ ^]^ Considering the symmetry‐breaking principle, alloying could be as a potential approach to induce nontrivial topological spin textures. First, incorporation of a second magnetic metal element can destroy the inherent inversion symmetry. Second, the additional spin lattice of alloying element can readily tune the exchange interaction and magnetic anisotropy. In a pioneering study, room‐temperature Néel‐type skyrmions were recently realized in 50% Co‐doped Fe_5_GeTe_2_.^[^ ^36^ ^]^ Therefore, it is imperative to clarify the effect of alloying magnetic elements on the formation of AFM skyrmions in the emerging fields of 2D magnets.

In this work, we have theoretically proposed an alloying strategy to trigger AFM skyrmions in an AFM NiPS_3_ monolayer. At the 2D limit, NiPS_3_ was found to be an antiferromagnet with XY‐type magnetic ordering. The alloying element Cr was selected from a large number of transition metals by comparing the bond length, magnetic ground state, exchange interaction, magnetic anisotropy energy (MAE) and DMI. Using first‐principles calculations and Monte‐Carlo simulations, we found that a sizable DMI can be induced in alloyed Ni_1‐_ *_x_* Cr*_x_* PS_3_ (*x* = 1/4, 1/2, and 3/4) monolayers. Consequently, Ni_1‐_ *_x_* Cr*_x_* PS_3_ monolayers exhibit chiral magnetic states of AFM skyrmions in the case of *x* = 1/4. The skyrmions are <12 nm in diameter and can be sustained up to an external magnetic field of 4 T. In addition to the DMI, the competing magnetic constants of Heisenberg exchange coupling and magnetic anisotropy were also discussed to clarify the underlying mechanism of the observed complex spin texture. Our results suggest that alloying is an effective strategy to modulate the topological spin textures of 2D antiferromagnets, highlighting promising applications in skyrmion‐based spintronics.

## 2 Results and Discussion

**Figure 1:**
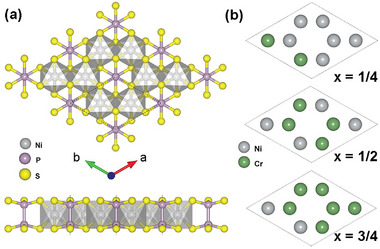
a) Top and side views of the crystal structure of monolayer NiPS_3_. The dotted lines represent the unit cell. b) The most stable alloying configurations of Ni_1‐_ *_x_* Cr*_x_* PS_3_ (*x* = 1/4, 1/2, and 3/4). Only magnetic atoms are shown here, with gray and green atoms representing Ni and Cr, respectively.

Previous experiments have demonstrated that 2D metal phosphorous trichalcogenides MPS_3_ are a new class of AFM semiconductors.^[^ ^13^, ^37^ ^]^ The monolayer structure of MPS_3_ consists of octahedral MS_6_ units with the layer group $p\bar{3}1m$. Similar to the 1T phase of 2D MoS_2_, MPS_3_ can be viewed as 1/3 of the M atom in MS_2_ being replaced by P_2_ dimers, corresponding to an equivalent stoichiometry of M_2/3_(P_2_)_1/3_X_2_. As shown in **Figure** **1**a, the (P_2_S_6_)^4−^ anion is located at the center of the honeycomb lattice by M atoms. In experiments, the M cations in the synthetic MPS_3_ monolayer materials are magnetic 3*d* transition metals, including Fe, Mn, Ni, and Co. Depending on the species of magnetic ions M in the 2D host lattice, the AFM ordering and critical Néel temperature (T_N_) varies from Ising‐type for FePS_3_ (T_N_ = 123 K), to Heisenberg‐type for MnPS_3_ (T_N_ = 78 K), to XY‐type for NiPS_3_ (T_N_ = 155 K) and XY‐type for CoPS_3_ (T_N_ = 122 K).^[^ ^38^ ^]^ It is also known that in‐plane magnetic anisotropy and high magnetic transition temperature are crucial for the formation of topological spin textures.^[^ ^34^, ^35^ ^]^ Therefore, NiPS_3_ monolayer was selected as a model compound for alloying to achieve the desired topological spin textures.

To investigate the influence of the alloying elements on the magnetic textures of NiPS_3_ monolayer, we systematically calculated the structural parameters, electronic properties, magnetic interaction, and MAE of pristine NiPS_3_ monolayer. As listed in Table S1 (Supporting Information), the optimized lattice parameters (a = 5.81 Å) and bandgap (E_g_ = 1.72 eV) of NiPS_3_ monolayer are in good agreement with the experimental results (a = 5.82 Å, E_g_ = 1.6 eV).^[^ ^39^, ^40^ ^]^ To confirm the magnetic ground state of NiPS_3_, we compared the total energies of the FM state and three kinds of AFM states (Néel‐type, Stripy‐type, and Zigzag‐type). Their spin configurations are displayed in Figure S1 (Supporting Information). We found that the Zigzag‐type AFM is the lowest‐energy state, whose energy is 82.7 meV/formula lower than that of the FM state.

We further adopted a Heisenberg spin Hamiltonian to describe the magnetic interactions in NiPS_3_ monolayer:

(1) $$H=-\sum_{\langle i,j\rangle }J_{\mathrm{ij}}\left(S_{i}\cdot S_{j}\right)-\sum_{\langle i,j\rangle }D_{\mathrm{ij}}\cdot \left(S_{i}\times S_{j}\right)-\sum_{i}K_{i}{\left(S_{i}^{z}\right)}^{2}$$

The first and second terms represent symmetric and antisymmetric parts of the exchange couplings, respectively. *S_i_* and *S_j_* are the spins of the *i* and *j* sites. *J_ij_* and *D_ij_* are the Heisenberg isotropic exchange coefficients and DMI strength between spins *S_i_* and *S_j_*, respectively. *K* is the single‐ion anisotropy coefficient, *S_i_^z^* is the *z* component of the spin at position *i*. The magnetic parameters *J*, *D*, and *K* are calculated by considering four specifically designed non‐collinear spin configurations according to the well‐established four‐state method.^[^ ^41^, ^42^ ^]^

**Figure 2:**
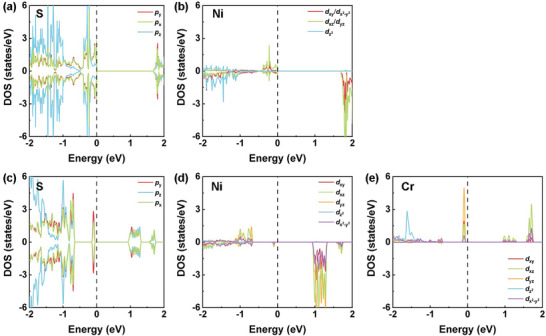
Projected density of states (PDOS) of magnetic atoms of Ni_1‐_ *_x_* Cr*_x_* PS_3_ monolayers. PDOS of a) S and b) Ni of NiPS_3_. PDOS of c) S, d) Ni, and e) Cr of Ni_3/4_Cr_1/4_PS_3_.

**Figure 3:**
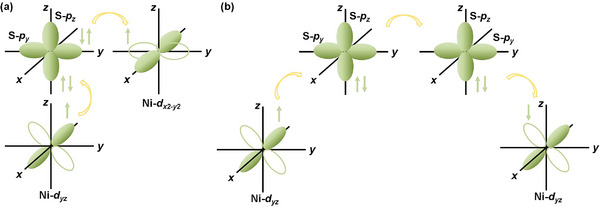
The schematic diagrams of a) superexchange paths of Ni‐*d_yz_*, S‐*p_z_*/*p_y_*, and Ni‐*d_x2‐y2_*, b) super‐superexchange paths of Ni‐*d_yz_*, S‐*p_z_*/*p_y_*, S‐*p_y_*/*p_z_* and Ni‐*d_yz_* in NiPS_3_.

The calculated exchange coupling parameters for NiPS_3_ monolayer are *J*_1_ = 1.6 meV, *J*_2_ = 0.36 meV, and *J*_3_ = −7.45 meV, consistent with the experimental values.^[^ ^43^ ^]^ These results reveal FM *J*_1_ and *J*_2_ as well as a larger AFM *J*_3_, which are mainly determined by the Goodenough‐Kanamori‐Anderson (GKA) rules.^[^ ^44^ ^]^ The magnetic Ni atoms are coordinated with six S atoms, forming an anti‐triangular prism of NiS_6_. Under the corresponding crystal field, five Ni‐3*d* orbitals split into two‐fold degenerate e_1_ (*d_xz_* + *d_yz_*), e_2_ (*d_x2‐y2_* + *d_xy_*) and a single a_1_ (*d_z2_*) states. For the first nearest neighbors, there is no direct Ni‐Ni exchange since the corresponding overlapping orbitals for Ni^2+^ are filled. The near‐90° Ni‐S‐Ni superexchange interaction (with Ni‐S distance of 2.44 Å) contributes to the FM interaction of the first nearest Ni atoms. According to the projected density of states (PDOS) of NiPS_3_ monolayers (**Figure** **2**a,b), a near‐90° superexchange interaction between spin‐up occupied Ni‐*d_yz/xz_* orbital and S‐*p_z_*/*p_x/y_* orbitals and spin‐down unoccupied Ni‐*d_x2‐y2_* orbital leads to weak ferromagnetism. For the second nearest Ni atoms, there is neither direct exchange path. The superexchange between the M1 and M3 Ni atoms occurs via two S atoms from different layers (Figure S2, Supporting Information). Therefore, the *J*_2_ value is positive and very small. For the third nearest exchange interaction, the super‐superexchange path M1‐S1…S5‐M4 is activated because two S atoms are in the same layer. The Ni‐S and S‐S distances are 2.44 and 3.47 Å, respectively, and the Ni‐S‐Ni angle is 131°. Based on the PDOS of NiPS_3_, the super‐superexchange interaction arises from the electrons hopping between spin‐up occupied Ni‐*d_yz/xz_* orbital and S‐*p_z_*/*p_x/y_* orbitals, resulting in the antiferromagnetism. According to the threefold rotation operation symmetry, the third neighbor hopping is dominant in 2D NiPS_3_.^[^ ^45^ ^]^ As a consequence, *J*_3_ is negative and larger than the other exchange parameters, and the magnetic ground state of NiPS_3_ is Zigzag‐type AFM. To further illustrate the process of electron hopping, the schematic diagrams of Ni‐S‐Ni superexchange and Ni‐S…S‐Ni super‐superexchange are displayed in **Figure** **3**a,b.

**Figure 4:**
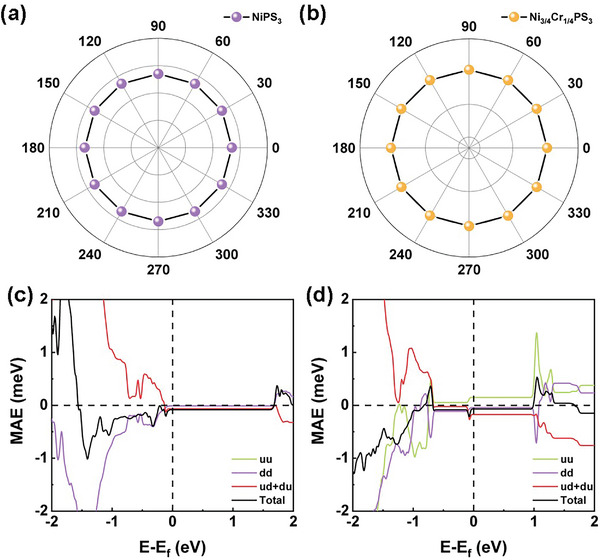
Angular dependence of MAEs of a) NiPS_3_, b) Ni_3/4_Cr_1/4_PS_3_ monolayer with magnetization direction lying on XY planes. Fermi level dependent decomposed and total MAEs of c) NiPS_3_, d) Ni_3/4_Cr_1/4_PS_3_ monolayer. The uu, dd, and ud+du represent the spin coupling between the spin‐up channels, spin‐down channels, and spin‐up with spin‐down channels, respectively. The Fermi level is set to zero.

Another important physical parameter is the MAE, which is defined as the difference between ground‐state energies due to the rotation of magnetization direction. To determine the easy axis, MAE is calculated as follows:

(2) $$MAE\;=E_{\theta }\;-E_{001}$$

where *E*_θ_ and *E*_001_ refer to the total energies of the states whose magnetization direction lies in the XY plane with the angle *θ* and perpendicular to the XY plane, respectively. For NiPS_3_ monolayer, the calculated MAE is −82.7 µeV per magnetic atom, meaning that the easy magnetization orientation is parallel to the XY plane. **Figure** **4**a plots the angular dependence of MAE in the XY plane. One can see that MAE in the XY plane is nearly isotropic, which coincides the experimental observation that NiPS_3_ monolayer is a 2D XY‐type antiferromagnet.^[^ ^46^ ^]^ To further elucidate the origin of MAE, we decomposed the MAE of NiPS_3_ into three coupling terms using the torque procedure,^[^ ^47^ ^]^ namely the majority spin states (uu), minority spin states (dd), and cross spin states (ud+du), with respect to the Fermi level (Figure 4c). It can be seen that the ud+du channel makes the largest contribution to the in‐plane MAE. Combining the PDOS of Ni atoms in Figure 2a,b, one can conclude that the negative MAE of NiPS_3_ mainly originates from the ud+du coupling channel between spin‐up occupied Ni‐*d_xz_* orbital and spin‐down unoccupied Ni‐*d_yz_* orbital, as well as spin‐up occupied Ni‐*d_x2‐y2_* and spin‐down unoccupied Ni‐*d_xy_* orbital. On the other hand, the inherent inversion symmetry of 2D NiPS_3_ leads to the absence of DMI.

Based on the aforementioned magnetic behavior of 2D NiPS_3_, the general principles for modulating the magnetic skyrmions in 2D AFM materials can be summarized as follows: i) breaking the inherent symmetry and inducing a large DMI; ii) moderately weakening the Heisenberg exchange interactions by increasing the distance between transition metal ions or that between transition metal and non‐metal bridge ions; iii) achieving a larger in‐plane MAE by weighting the contributions of SOC interactions between relevant coupling terms. Based on the above analysis, we first considered five elements of 3*d* magnetic transition metals (M = V, Cr, Mn, Fe, Co). Taking 1:1 ratio of Ni:M as an example, we further filtered the alloying element by calculating the bond lengths, magnetic ground state, *J*, DMI, *D*/|*J*|, and MAE of Ni_1/2_M_1/2_PS_3_. The detailed results are summarized in Table S2 (Supporting Information). After alloying, the resulting five Ni_1/2_M_1/2_PS_3_ systems exhibit different magnetic ground states, that is, Ni_1/2_Cr_1/2_PS_3_, Ni_1/2_Mn_1/2_PS_3_, and Ni_1/2_Co_1/2_PS_3_ are still in AFM state like NiPS_3_, while FM and ferrimagnetic states are energetically more favorable in Ni_1/2_V_1/2_PS_3_ and Ni_1/2_Fe_1/2_PS_3_, respectively. Compared with pristine NiPS_3_ (*J*_1_ = 1.63 meV, *J*_2_ = 0.36 meV, *J*_3_ = −7.45 meV), the magnetic exchange coupling strengths are systematically weakened. For Ni_1/2_V_1/2_PS_3_ and Ni_1/2_Cr_1/2_PS_3_, *J*_1_ is reduced to 0.3 and 1.25 meV, *J*_2_ is reduced to 0.079 and 0.25 meV, *J*_3_ is reduced to 1.85 and −6.89 meV, respectively. Accordingly, sizable *D*/|*J*| is induced in alloy compounds of Ni_1/2_Cr_1/2_PS_3_ (26.7%) and Ni_1/2_Co_1/2_PS_3_ (59.9%). The calculated magnetic anisotropy still favors in‐plane magnetization for V, Cr, Mn, and Co, but out‐of‐plane for Fe. Among them, the largest anisotropy energy barrier (77.3 µeV) between in‐plane and out‐of‐plane direction is found in Ni_1/2_Cr_1/2_PS_3_, which is ≈7, 14, and 22 times higher than that of Ni_1/2_Mn_1/2_PS_3_, Ni_1/2_Co_1/2_PS_3_, and Ni_1/2_V_1/2_PS_3_, respectively. Therefore, Cr is chosen as the alloying element for the successive discussions about topological magnetic texture.

To further investigate Cr alloying‐induced topological spin switching, we constructed a series of model structures of Ni_1‐_ *_x_* Cr*_x_* PS_3_ by varying Cr/Ni compositions, i.e., *x* = 1/4, 1/2, 3/4. For each concentration, we considered all possible six structurally ordered phases within a 2×2 supercell (Figure S3a–c, Supporting Information). These ordered phases have a definite structure and have been shown to be more stable than the disordered phases.^[^ ^48^, ^49^ ^]^ By comparing the relative energies (Figure S3d–f, Supporting Information), we finally identified the most stable structures for different Ni_1‐_ *_x_* Cr*_x_* PS_3_ monolayers (Figure 1b). The equilibrium lattice parameters, bond lengths and bond angles of Ni_1‐x_Cr_x_PS_3_ are listed in Table S1 (Supporting Information). It can be seen from the Table S1 (Supporting Information) that the larger ionic radius of Cr compared to Ni leads to a gradual increase in the lattice constant of the structure and a disruption of the original crystal symmetry as the alloyed Cr content increases,

These structures were then utilized to calculate the stability, electronic and magnetic properties. In order to characterize the energetics for alloying different Cr concentrations in the host NiPS_3_ monolayer, we calculated the formation energies defined as

(3) $$E_{f}=\left[E_{Ni_{1-x}Cr_{x}PS_{3}}\;-\left(1-x\right)E_{Ni}-xE_{Cr}-E_{P}-3E_{S}\right]/5$$

where $E_{Ni_{1-x}Cr_{x}PS_{3}}$ is the energy of Ni_1‐_ *_x_* Cr*_x_* PS_3_ monolayers per formula, and *E*_Ni_, *E*_Cr_, *E*_P_, and *E*_S_ are the energy per atom of Ni, Cr, P, and S elements in their most stable solid states, respectively. By definition, a negative *E_f_* value indicates that formation of Ni_1‐_ *_x_* Cr*_x_* PS_3_ monolayer is exothermic. The calculated *E_f_* of Ni_1‐_ *_x_* Cr*_x_* PS_3_ monolayers are −0.54 eV/atom (*x* = 1/4), −0.68 eV/atom (*x* = 1/2), and −0.83 eV/atom (*x* = 3/4), respectively. They are all more stable than the pristine NiPS_3_, whose *E_f_* is −0.41 eV/atom. In addition, ab initio molecular dynamics (AIMD) simulations at 300 K were performed for these Ni_1‐_ *_x_* Cr*_x_* PS_3_ monolayers. As shown in Figure S4 (Supporting Information), there are no significant lattice deformation after 10 ps simulation, indicating satisfactory thermal stability.

The preferred magnetic ground states of Ni_1‐_ *_x_* Cr*_x_* PS_3_ monolayers were confirmed by comparing the energies of FM state and various possible AFM configurations, whose spin densities are displayed in Figure S5 (Supporting Information). As shown in Table S3 (Supporting Information), all these systems prefer AFM as the magnetic ground states, which have lower energy than the FM state by 53.9 (*x* = 1/4), 54.3 (*x* = 1/2), 40.2 (*x* = 3/4) meV/formula, respectively. The projected electronic band structures revealed that all Ni_1‐_ *_x_* Cr*_x_* PS_3_ monolayers are semiconductors with moderate band gaps of 1.04 eV (*x* = 1/4), 0.94 eV (*x* = 1/2) and 0.98 eV (*x* = 3/4), respectively, as shown in Figure S6b–d (Supporting Information). As expected, the local magnetic moments in these Ni_1‐_ *_x_* Cr*_x_* PS_3_ monolayers are mainly contributed by the *d* orbitals of Ni and Cr atoms, i.e., 1.4 µ_B_ on Ni and 3.8 µ_B_ on Cr.

**Figure 5:**
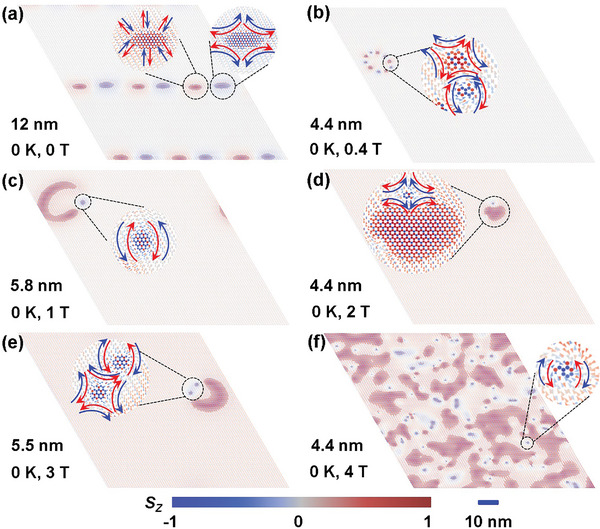
Top views of the real‐space distribution of magnetic moments from snapshots of MC simulations under different out‐of‐plane magnetic fields for Ni_3/4_Cr_1/4_PS_3_ monolayer. The color map represents the out‐of‐plane spin component of the magnetic atoms. The insets show the enlarged image of AFM skyrmions.

For these AFM semiconductors Ni_1‐_ *_x_* Cr*_x_* PS_3_ (*x* = 1/4, 1/2, 3/4) monolayers, the calculated magnetic parameters *J*, *D*, and MAE are listed in Table S4 (Supporting Information). With these parameters from first‐principles calculations, MC simulations were carried out to investigate the possible topological spin textures in Ni_1‐_ *_x_* Cr*_x_* PS_3_ monolayers. As shown in **Figure** **5**a, it is surprisingly to found spontaneous AFM skyrmions in Ni_3/4_Cr_1/4_PS_3_ monolayers without an external magnetic field. This unique spin texture was not observed for the other two concentrations (as shown in Figure S7, Supporting Information). In particular, the diameter of AFM skyrmions in the Ni_3/4_Cr_1/4_PS_3_ monolayer is 12 nm at zero field. Such a small size is urgently needed for both experimental and theoretical works. In addition, the topological charge (*Q*) is a crucial parameter describing topological properties, which is defined as, $Q\;=\frac{1}{4\pi }\;\int S\cdot \left(\partial_{x}S\times \partial_{y}S\right)dxdy.$ ^[^ ^50^ ^]^ The calculated topological charge *Q*  =  0, which is consistent with the typical topological charge of AFM skyrmions.^[^ ^26^ ^]^ This result stems from the composition of the AFM skyrmions, which consist of two similar but opposite sublattices.

To unveil the underlying mechanism of intrinsic AFM skyrmions due to substituting 25% Ni with Cr, we first analyzed the alloying effect on the magnetic exchange coupling strength *J*. The calculated *J*_1_, *J*_2_ and *J*_3_ of Ni_3/4_Cr_1/4_PS_3_ monolayers are listed in Table S4 (Supporting Information). For the exchange interaction between the first nearest neighbors, there are two new possible electron hopping paths introduced by alloying Cr, namely, the near‐90° superexchange between the Cr‐S‐Ni and the direct spin exchange between Ni‐Cr (the Ni‐Cr distance is 3.21 Å). Due to the larger ionic radius of Cr, the distance of the electron hopping path becomes larger (the Ni‐S and Cr‐S distance are 2.44 and 2.54 Å, respectively). Based on PDOS in Figure 2c–e, a near 90° superexchange interaction between spin‐up occupied Cr‐*d_yz_* and S‐*p_z_*/*p_y_* orbitals and spin‐down unoccupied Ni‐*d_x2‐y2_* orbitals gives rise to weak ferromagnetism. The direct exchange between the occupied Ni‐*d_x2‐y2_* and the occupied Cr‐*d_xy_* orbitals leads to antiferromagnetism. The calculated *J*_1_ parameters between Ni‐Ni and Ni‐Cr pairs are 1.44 and 1.32 meV, respectively. Compared to pristine 2D NiPS_3_, the weakened FM couplings between Ni‐*d_yz_*, Ni‐*d_x2‐y2_* and S‐*p_z_*/*p_y_*, as well as the competition between antiferromagnetism of Ni‐Cr direct exchange and ferromagnetism of Cr‐S‐Ni superexchange lead to the observation that the alloying Cr atom has strong influence on the weakening of FM coupling between first nearest neighbors. For the second nearest neighbor, the long‐distance exchange interactions of Ni‐Ni, Ni‐Cr. and Cr‐Cr pairs can alternatively produce FM and AFM ordering, but these are rather negligible owing to very small *J*_2_ values (−0.22, 0.30, and −0.12 meV). For the third nearest neighbor exchange interaction, there are mainly two possible electron hopping paths, i.e., Ni‐S…S‐Ni and Cr‐S…S‐Ni. Based on the PDOS in Figure 2c–e, electron hopping between spin‐up occupied Cr‐*d_yz/xz_* orbital and S‐*p_z_*/*p_x/y_* orbitals and S‐*p_x/y_*/*p_z_* and spin‐up occupied Ni‐*d_yz/xz_* orbital favors antiferromagnetism. The enhanced coupling between Ni‐*d_yz_* and Ni‐*d_xz_* with S‐*p_z_*/*p_y_* leads to enhanced antiferromagnetism (Table S4, Supporting Information). Although the magnetic ground state of Ni_3/4_Cr_1/4_PS_3_ monolayer is still AFM, the incorporation of the second magnetic element Cr provides some additional electron hopping pathways. As a result, *J*_1_ and *J*_2_ are reduced with regard to pristine NiPS_3_, while *J*_3_ is enhanced.

For Ni_3/4_Cr_1/4_PS_3_ monolayer, the angular dependence of MAE along the XY plane was calculated and plotted in Figure 4b. Similar to NiPS_3_ monolayer, Ni_3/4_Cr_1/4_PS_3_ system also exhibits in‐plane XY type magnetic ordering. The energy barrier of magnetic easy axis along the XY plane and perpendicular to the XY plane is −70.1 µeV per magnetic atom, which is slightly lower than that of NiPS_3_ monolayer (−82.7 µeV). To further analyze the relationship between MAE and Cr content, we decomposed the MAE of Ni_3/4_Cr_1/4_PS_3_ monolayer into three coupling terms with respect to the Fermi level (Figure 4d). It can be seen that the large negative MAE originates mainly from the strong coupling through the ud+du channels. Combining PDOS of *d* orbitals of the magnetic atoms in Figure 2c–e, the coupling orbitals are still spin‐up occupied Ni‐*d_yz_* and spin‐down unoccupied Ni‐*d_xz_*. Moreover, the contribution of uu coupling channel between spin‐up occupied Cr‐*d_yz_* and spin‐up unoccupied Cr‐*d_xz_* is enhanced in Ni_3/4_Cr_1/4_PS_3_ monolayer, giving rise to a positive MAE. This contribution lowers the total MAE to a smaller negative value.

The inclusion of a second transition metal element not only modulates the amplitudes of exchange coupling *J* and MAE, but also destroys the inherent inversion symmetry of NiPS_3_. Therefore, a nonzero DMI value is generated by Cr alloying. In Ni_3/4_Cr_1/4_PS_3_ monolayer, the induced DMI values (*D*_1_) are 0.017 meV between the first nearest neighbor Ni‐Ni and 0.054 meV between Ni‐Cr, respectively. The *D*_2_ values are 0.009, 0.007, and 0.001 meV for second nearest neighbors Ni‐Ni, Ni‐Cr, and Cr‐Cr, respectively, and *D*_3_ are 0.007 and 0.002 meV for the third nearest neighbors Ni‐Ni and Ni‐Cr, respectively. The DMI amplitudes of Ni_3/4_Cr_1/4_PS_3_ monolayer decrease rapidly with increasing distance between magnetic ions and are mainly dominated by the Cr ions. It is important to note that the current DMI is smaller than those of 2D CrInSe_3_,^[^ ^22^ ^]^ Tl_2_NO_2_,^[^ ^51^ ^]^ and MnSTe,^[^ ^24^ ^]^ which can be explained by the Fert‐Levy mechanism of DMI.^[^ ^52^ ^]^ Compared to S in Ni_3/4_Cr_1/4_PS_3_ compound, the heavier non‐magnetic atoms (Se and Te), act as spin‐orbit active sites and induce more significant spin‐orbit scattering, ultimately leading to a larger DMI. Similar behavior has been discovered for the MnXY (X, Y = S, Se, Te)^[^ ^24^ ^]^ and CrInX_3_ (X = Se, Te).^[^ ^22^ ^]^ The *D*_1_/|*J*_1_| ratio between DMI and the exchange parameter is 1.21% and 4.1% for Ni‐Ni and Ni‐Cr, respectively. Recently, it has also been shown that such amplitude of *D*/|*J*| ratio can induce topological spin textures in 2D AgCr_2_X_4_ (X = S or Se).^[^ ^53^ ^]^ In Ni_3/4_Cr_1/4_PS_3_ monolayer, the weak anisotropy of XY‐type magnetic ordering may be beneficial for the formation of skyrmions without large *D*/|*J*|.

To confirm this, we also calculate the angular dependence of MAE along the XY plane of Ni_1/2_Cr_1/2_PS_3_ and Ni_1/4_Cr_3/4_PS_3_ monolayers, which don't have the properties of AFM skyrmions. The results are shown in Figure S8 (Supporting Information) and one can observe a strong in plane MAE anisotropy. It is clear that the broken XY‐type magnetic ordering is not conductive to the formation of skyrmions. Therefore, the emergence of AFM skyrmions in 2D Ni_3/4_Cr_1/4_PS_3_ mainly relies on the enhanced DMI, suppressed *J*_1_ and unchanged XY‐type magnetic order during alloying. Based on the above factors, it is possible to extrapolate to other 2D magnetic semiconductor MPS_3_ layers. In particular, CoPS_3_ exhibits a significant potential to induce AFM skyrmions via alloying because it has a similar magnetic structure with NiPS_3_, which is also stabilized by the XY model. Moreover, Ni (1.24 Å) and Co (1.25 Å) also have a similar ionic radius.

Finally, we discuss the effects of magnetic field and temperature effect on the evolution of skyrmions. As can be seen in Figure 5b–f, the AFM skyrmions remain still visible under an external magnetic field up to 4 T. With increasing strength of the magnetic field, the diameter of the skyrmions generally decreases from 12 nm at 0 T to 4.4 nm at 4 T. The underlying reason for this phenomenon is that the external magnetic field promotes out‐of‐plane magnetization. The temperature effect on AFM skyrmions is depicted in Figure S9 (Supporting Information). The skyrmions remain clearly visible at 2 K. However, as the temperature increases to 5 K, the boundaries of the skyrmions become blurred, and they eventually disappear as the temperature further increases. Up to now, the skyrmion diameters reported experimentally are larger than 100 nm, which is an order of magnitude larger than the desired diameter (<10 nm) for memory applications.^[^ ^54^ ^]^ Our finding of small‐sized skyrmions is desirable for the synthesis of next‐generation memory devices with higher storage density.

## 3 Conclusion

To summarize, we theoretically propose a feasible alloying strategy to regulate the AFM skyrmions in 2D magnets based on symmetry considerations and analysis of exchange interactions. Starting from an AFM NiPS_3_ monolayer, a series of stable Ni_1‐_ *_x_* Cr*_x_* PS_3_ monolayers with high thermodynamic stability are predicted by first‐principles calculations. Monte‐Carlo simulations reveal that Ni_3/4_Cr_1/4_PS_3_ monolayer exhibits intrinsic AFM spin textures at zero field. When the external magnetic field is applied, the diameter of the skyrmions decreases from 12 to 4.4 nm and the skyrmion phase can be retained up to an external field of 4 T. The emergence of AFM skyrmions is attributed to the alloying Cr element, which induces considerable DMI, suppresses the exchange coupling strength, and maintains the weak easy‐plane (XY plane) magnetic anisotropy. All these results demonstrate that alloying might be an effective way to induce topological spin textures in 2D magnets. Hence, further comprehensive experimental and theoretical investigations are desired to substantiate this pivotal argument.

## 4 Experimental Section

The spin‐polarized density functional theory calculations were performed with the Vienna Ab Initio Simulation Package.^[^ ^55^ ^]^ The projector augmented wave ^[^ ^56^ ^]^ method was used for ion‐electron interactions and the Perdew–Burke–Ernzerhoffunctional within the generalized gradient approximation (GGA)^[^ ^57^ ^]^ for exchange‐correlation interactions. The criteria for energy and force convergences were 10^−6^ eV and 10^−3^ eV Å^−1^, respectively. The cutoff energy of the plan‐wave basis was 600 eV. To minimize the interaction between neighboring images, a vacuum region of 20 Å was applied along the z‐direction. A Γ‐centered Monkhorst–Pack *k*‐point grid with a uniform spacing of 0.02 Å^−1^ was used for sampling the Brillouin zone. Considering the strong correlation effect of *d* electrons, GGA plus on‐site repulsion U method with an effective Coulomb parameter *U*_eff_ = 4 eV was adopted for the *d* orbitals of Ni and Cr.^[^ ^58^ ^]^ To investigate the spin dynamics of Ni_1‐_ *_x_* Cr*_x_* PS_3_ monolayers, Monte Carlo simulations were performed using the Metropolis algorithm implemented in the *Spirit* package.^[^ ^59^ ^]^ An 80 × 80 × 1 periodical supercell with 51200 spin sites was used to simulate the evolution of spin textures. For each temperature and magnetic field strength, at least 7 × 105 MC steps were simulated.

## Conflict of Interest

The authors declare no conflict of interest.

## Supporting information

Supporting Information

## Data Availability

The data that support the findings of this study are available from the corresponding author upon reasonable request.
