# Supplementary material for: Alloying Driven Antiferromagnetic Skyrmions on NiPS3 Monolayer: A First‐Principles Calculation
Source: Adv Sci (Weinh). 2024 Apr 22;11(25):2401048. doi: 10.1002/advs.202401048 (PMC11220710; doi:10.1002/advs.202401048)
Supplement: Supplementary file 1 — Supporting Information [file ADVS-11-2401048-s001.pdf]

ADVANCED SCIENCE
Open Access

Supporting Information

for *Adv. Sci.*, DOI 10.1002/advs.202401048

Alloying Driven Antiferromagnetic Skyrmions on $NiPS_3$ Monolayer: A First-Principles Calculation

Yanxia Wang, Jianpei Xing, Ying Zhao, Yi Wang, Jijun Zhao and Xue Jiang**

Supporting Information for

Alloying Driven Antiferromagnetic Skyrmions on $NiPS_3$ Monolayer: A First-principles Calculation

Yanxia Wang[1], Jianpei Xing[1], Ying Zhao[1], Yi Wang[1], Jijun Zhao[1,2,3*], Xue Jiang[1,2,3*]

[1]*Key Laboratory of Materials Modification by Laser, Ion and Electron Beams, Dalian University of Technology, Ministry of Education, Dalian 116024, China*

[2]*Guangdong Provincial Key Laboratory of Quantum Engineering and Quantum Materials, School of Physics, South China Normal University, Guangzhou 510006, China*

[3]*Guangdong-Hong Kong Joint Laboratory of Quantum Matter, Frontier Research Institute for Physics, South China Normal University, Guangzhou 510006, China*

Table S1. The optimized and experimental structure parameters of $Ni_{1-x}Cr_xPS_3$. The lattice parameters are a and b. d_{M-S}, d_{P-S}, d_{P-P} and ∠M-S-M represent the bond length and bond angle.

	**a (Å)**	**b (Å)**	**d_{M-S} (Å)**	**d_{P-S} (Å)**	**d_{P-P} (Å)**	**∠M-S-M**	**E_g (eV)**
**$NiPS_3$ (opt)**	5.81	5.81	2.44	2.05	2.18	86.74°	1.73
**$NiPS_3$ (exp)**	5.82	5.82	2.50	1.98	2.17	84.4°	1.6
**$Ni_{3/4}Cr_{1/4}PS_3$**	11.73	5.90	2.44/2.54	2.05	2.20	86.49°	1.04
**$Ni_{1/2}Cr_{1/2}PS_3$**	11.80	5.97	2.50/2.73	2.06	2.22	84.46°	0.94
**$Ni_{1/4}Cr_{3/4}PS_3$**	11.91	5.95	2.51/2.84	2.07	2.20	80.13°	0.98

* Corresponding author. E-mail: zhaojj@dlut.edu.cn (Jijun Zhao); jiangx@dlut.edu.cn (Xue Jiang)

Table S2. The magnetic and electronic properties of $Ni_{1/2}M_{1/2}PS_3$ (M = 3*d* magnetic transition element). GS, $d_{M\text{-}S}$, E_g, MAE, J1 and D/J1 are the ground state, bond length between the metal and S, bandgap, magnetic anisotropy energy, the first neighbor exchange coupling parameter and ratio between the DMI and J, respectively.

Structure	GS	$d_{M\text{-}S}$ (Å)	E_g (eV)	MAE (μeV)	J1 (meV)	J2 (meV)	J3 (meV)	$\frac{D1}{J1}$	$\frac{D2}{J2}$	$\frac{D3}{J3}$
**$NiPS_3$**	AFM	2.44	1.73	−82.7	1.63	0.36	−7.77	/	/	/
**$Ni_{1/2}V_{1/2}PS_3$**	FM	2.59	1.54	−3.5	0.3	0.079	1.85	1.01%	2.42%	0.01%
**$Ni_{1/2}Cr_{1/2}PS_3$**	AFM	2.59	0.94	−77.3	−1.25	0.25	−21.45	26.7%	0.8%	0.03%
**$Ni_{1/2}Mn_{1/2}PS_3$**	AFM	2.62	1.72	−11.8	−1.73	−0.013	−6.89	1.25%	0.27%	0.05%
**$Ni_{1/2}Co_{1/2}PS_3$**	AFM	2.52	1.77	−5.5	−7.45	−9.96	−19.48	59.9%	28.7%	7.66%
**$Ni_{1/2}Fe_{1/2}PS_3$**	FIM	2.59	1.72	0.4	/	/	/	/	/	/

Table S3. The total energies relative to the FM states of the different magnetic configurations of $Ni_{1-x}Cr_xPS_3$. Energies are expressed in terms of meV/unit cell. FM, AFM and FIM represent the ferromagnetic, antiferromagnetic and ferrimagnetic states, respectively.

**Structure**	**FM**	**FIM**	**AFM1**	**AFM2**	**AFM3**
$\mathbf{Ni_{3/4}Cr_{1/4}PS_3}$	0	/	**-53.9**	-48.4	-51.2
$\mathbf{Ni_{1/2}Cr_{1/2}PS_3}$	0	31.6	**-57.0**	-54.3	/
$\mathbf{Ni_{3/4}Cr_{1/4}PS_3}$	0	/	-45.1	**-48.5**	-40.2

Table S4. Magnetic parameters of $Ni_{1-x}Cr_xPS_3$ (x = 1/4, 1/2, 3/4) between the first, second, and third nearest magnetic atoms pairs. J, D/|J| and MAE are Heisenberg exchange, the ratio between DMI and J, and magnetic anisotropy energy, respectively.

$Ni_{1-x}Cr_xPS_3$	J_1 (meV)	J_2 (meV)	J_3 (meV)	$\frac{D1}{J1}$	$\frac{D2}{J2}$	$\frac{D3}{J3}$	MAE (μeV)
**X=0**	1.62	0.36	-7.45	/	/	/	-82.7
**X=1/4**	1.44(Ni-Ni)<br>1.32(Ni-Cr)	-0.22(Ni-Ni)<br>0.30(Ni-Cr)<br>-0.12(Cr-Cr)	-8.34(Ni-Ni)<br>-1.54(Ni-Cr)	1.21%<br>4.10%	4.1%<br>2.5%<br>0.81%	0.1%<br>0.1%	-70.6
**X=1/2**	-1.25(Ni-Ni)<br>0.22(Ni-Cr)<br>-1.00(Cr-Cr)	0.25(Ni-Ni)<br>-0.40(Ni-Cr)<br>0.14(Cr-Cr)	-21.45(Ni-Ni)<br>0.247(Cr-Cr)	0.26%<br>26.7%<br>0.02%	0.8%<br>1.85%<br>0.1%	0.03%<br>0.17%	-77.3
**X=3/4**	1.54(Ni-Cr)<br>-1.44(Cr-Cr)	-21.63(Ni-Ni)<br>1.61(Ni-Cr)<br>3.34(Cr-Cr)	-17.67(Ni-Cr)<br>6.05(Cr-Cr)	4.66 %<br>0.25%	0.00%<br>0.10%<br>0.006%	0.02%<br>0.04%	-113.1

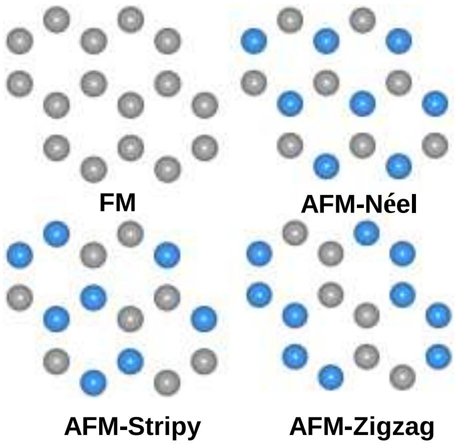

Figure S1. Four types of magnetic configurations of the $NiPS_3$, with one FM and three AFM. FM, AFM-Néel, AFM-Stripy, and AFM-Zigzag represent the ferromagnetic, Neel-type, Stripy-type and Zigzag-type antiferromagnetic states, respectively. Only magnetic atoms are shown here, where gray and blue atoms represent spin up and down, respectively.

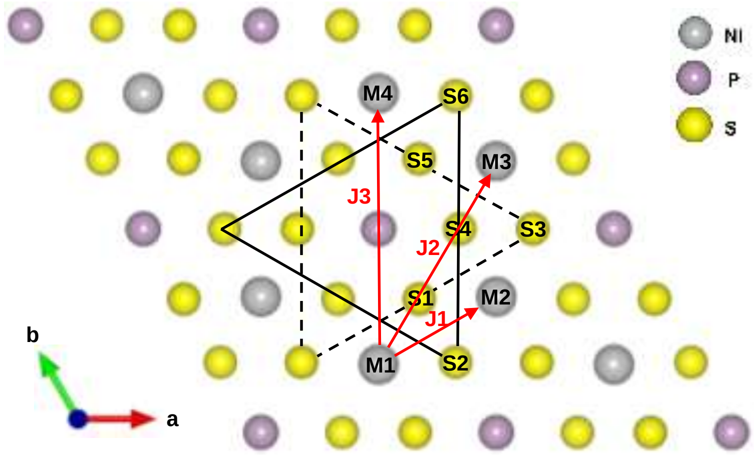

Figure S2. The schematic of the *ab* plane of $NiPS_3$ monolayer. The sulfur atomic layer above the *ab* plane is connected by a black solid line, and the sulfur atomic layer below the *ab* plane is connected by a black dotted line. The Ni atoms are marked as M1, …, M4, and sulfur atoms are labeled as S1, …, S6. J1, J2 and J3 represent the first, second and third nearest neighbors of magnetic atoms.

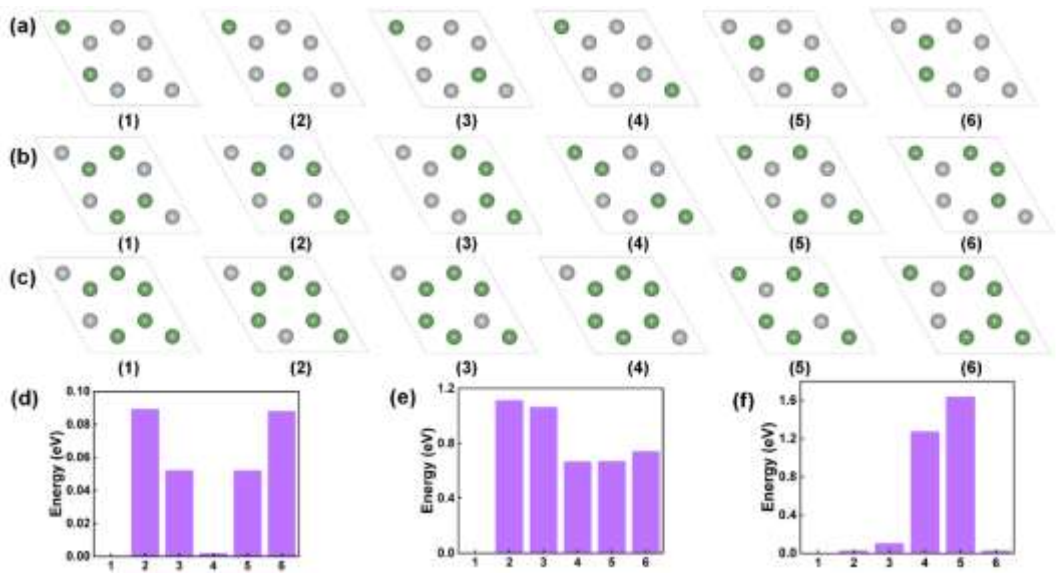

Figure S3. Considered possible alloying structures of Ni and Cr in 2×2 supercells for (a) $Ni_{3/4}Cr_{1/4}PS_3$, (b) $Ni_{1/2}Cr_{1/2}PS_3$, and (c) $Ni_{1/4}Cr_{3/4}PS_3$. Gray and green balls represent Ni and Cr atoms, respectively. The P and S atoms are omitted for simplicity. Relative energies per supercell of (d) $Ni_{3/4}Cr_{1/4}PS_3$, (e) $Ni_{1/2}Cr_{1/2}PS_3$, and (f) $Ni_{1/4}Cr_{3/4}PS_3$.

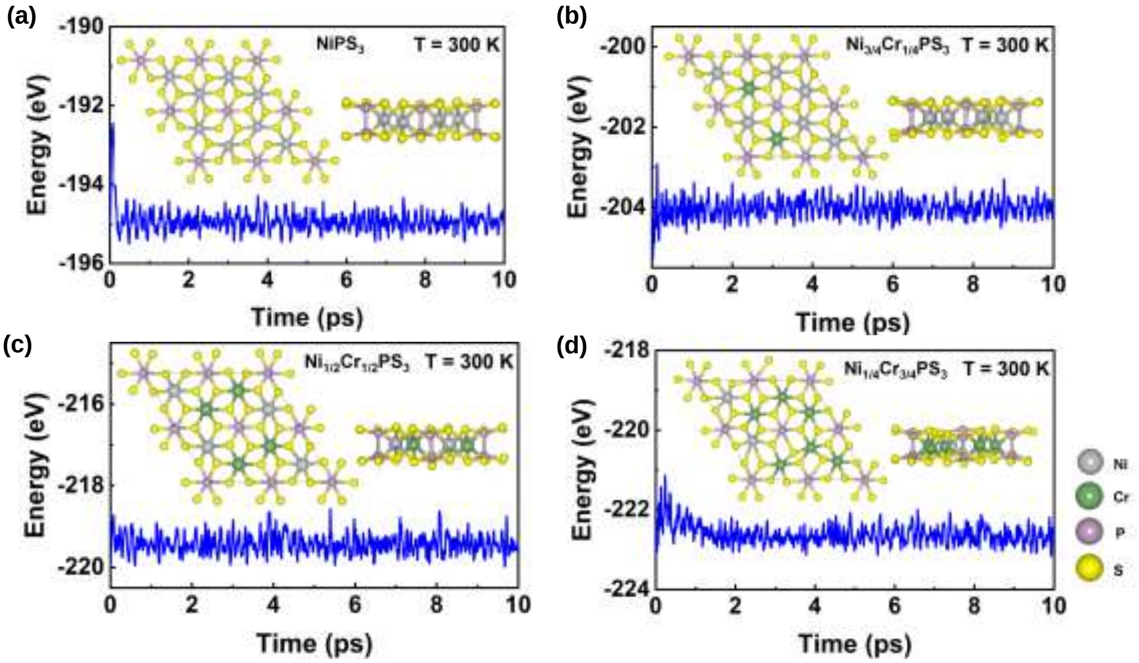

Figure S4. The total energy fluctuation for the (a) $NiCrPS_3$, (b) $Ni_{3/4}Cr_{1/4}PS_3$, (c) $Ni_{1/2}Cr_{1/2}PS_3$, and (d) $Ni_{1/4}Cr_{3/4}PS_3$ monolayers during AIMD simulations at 300 K. The illustrations show the top and side views of structures at the end of the simulations.

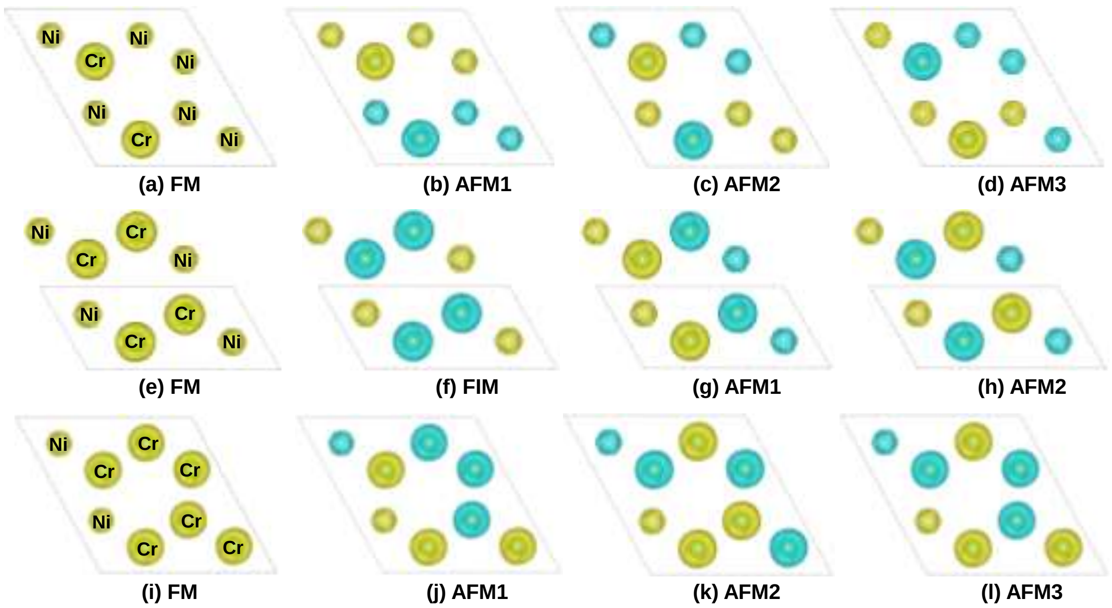

Figure S5. Spin density of the (a-d) $Ni_{3/4}Cr_{1/4}PS_3$, (e-h) $Ni_{1/2}Cr_{1/2}PS_3$, and (i-l) $Ni_{1/4}Cr_{3/4}PS_3$ monolayers. FM, AFM and FIM represent the ferromagnetic, antiferromagnetic and ferrimagnetic states, respectively. Yellow and blue density clusters represent spin up and spin down, respectively.

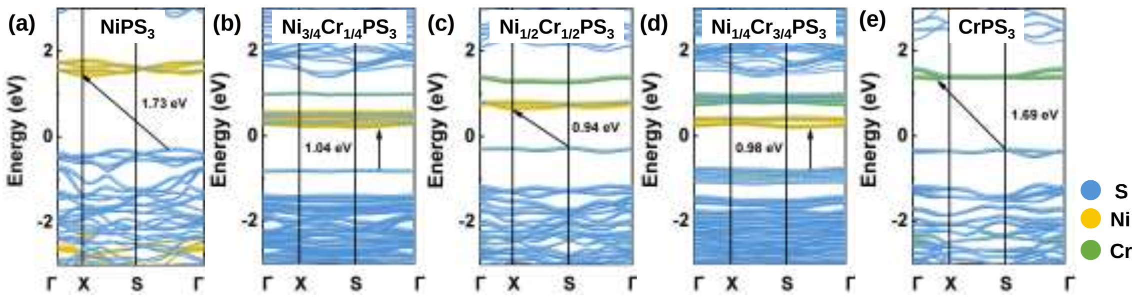

Figure S6. Atom-projected band structures of (a) $NiPS_3$, (b) $Ni_{3/4}Cr_{1/4}PS_3$, (c) $Ni_{1/2}Cr_{1/2}PS_3$, (d) $Ni_{1/4}Cr_{3/4}PS_3$ and (e) $CrPS_3$. The Fermi level is set to zero.

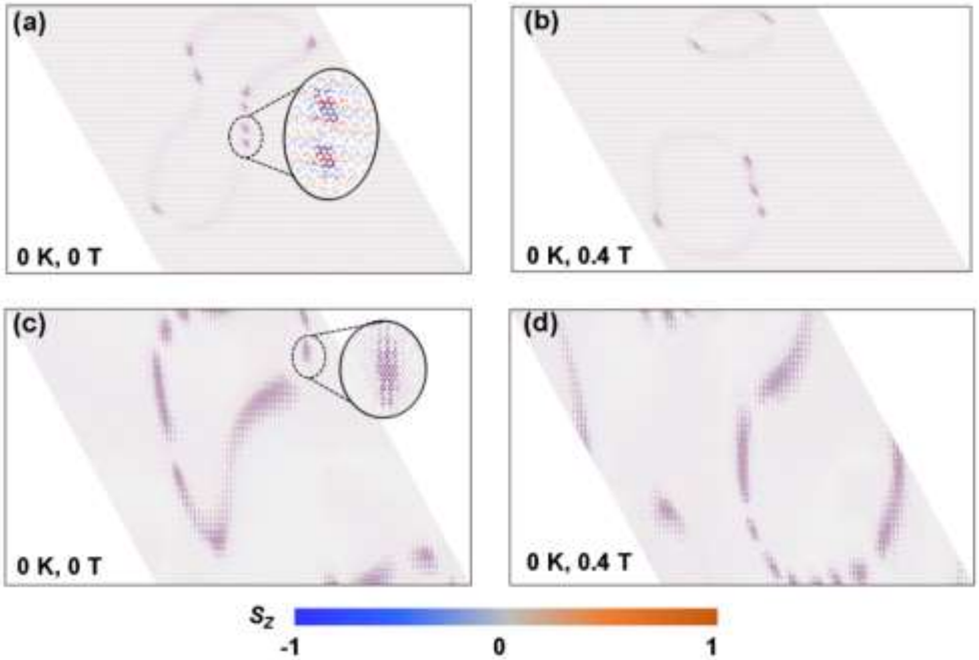

Figure S7. Top views of the real-space distribution of magnetic moments from snapshots of MC simulations under different out-of-plane magnetic fields for (a, b) $Ni_{1/2}Cr_{1/2}PS_3$ and (c, d) $Ni_{1/4}Cr_{3/4}PS_3$ monolayer.

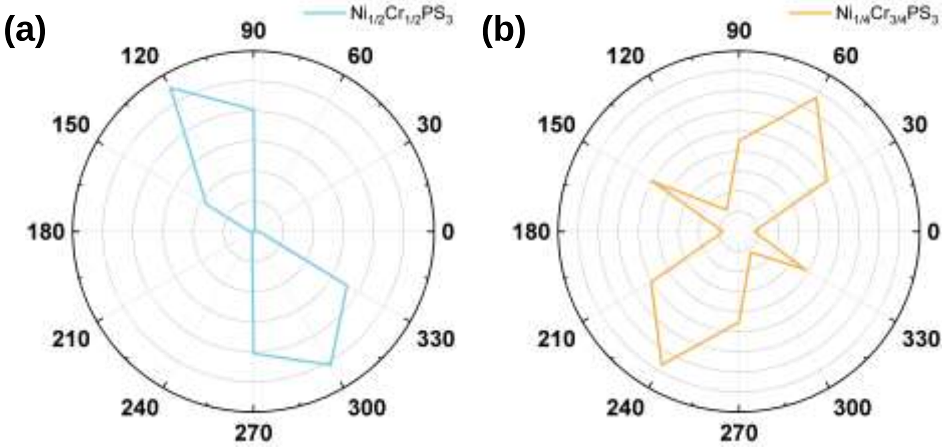

Figure S8. Angular dependence of MAEs of (a) $Ni_{1/2}Cr_{1/2}PS_3$, (b) $Ni_{1/4}Cr_{3/4}PS_3$ monolayer with magnetization direction lying on XY planes.

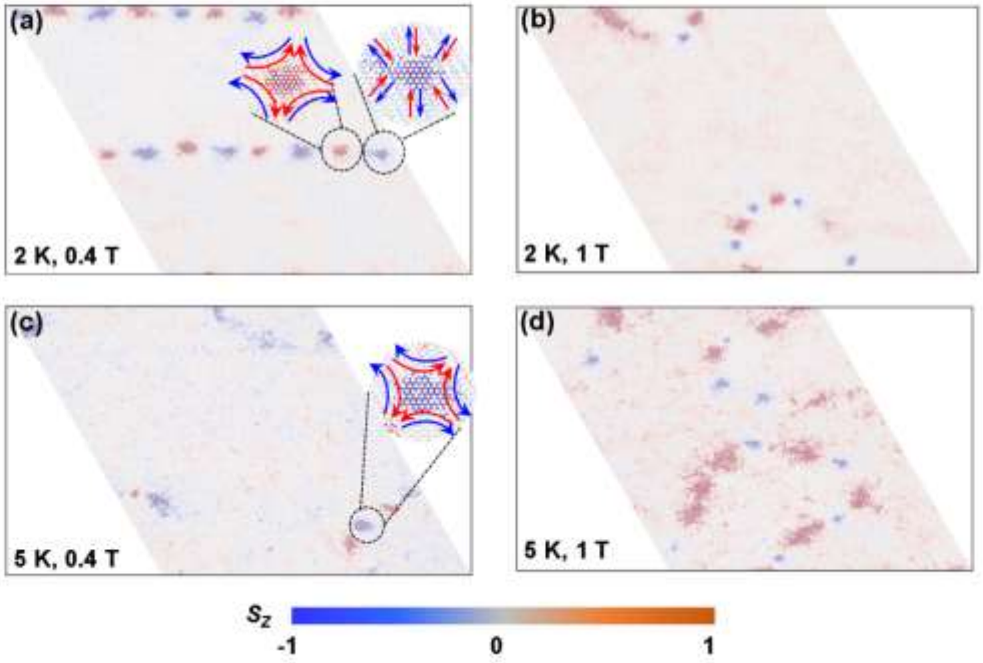

Figure S9. Top views of the real-space distribution of magnetic moments from snapshots of MC simulations under different temperatures and out-of-plane magnetic fields for $Ni_{3/4}Cr_{1/4}PS_3$ monolayers. The color map represents the out-of-plane spin component of the magnetic atoms. The insets show the enlarged image of antiferromagnetic skyrmions.
